# Precursors and preinvasive lesions of the breast: the role of molecular prognostic markers in the diagnostic and therapeutic dilemma

**DOI:** 10.1186/1477-7819-5-57

**Published:** 2007-05-31

**Authors:** Flora Zagouri, Theodoros N Sergentanis, George C Zografos

**Affiliations:** 1 Breast Unit, 1^st ^Department of Propaedeutic Surgery, Hippokratio Hospital, University of Athens, Athens, Greece

## Abstract

Precursors and preinvasive lesions of the breast include atypical ductal hyperplasia (ADH), ductal carcinoma in situ (DCIS), and lobular neoplasia (LN). There is a significant debate regarding the classification, diagnosis, prognosis and management of these lesions. This review article describes the current theories regarding the pathogenesis and molecular evolution of these lesions. It reviews the implication of a variety of molecules in the continuum of breast lesions: estrogen receptors (ER-alpha and ER-beta), c-erb-B2 (Her2/neu), p53, Ki-67, bcl-2, E-cadherin, transforming growth factor-beta (TGF-beta), p27 (Kip1), p16 (INK4a), p21 (Waf1), vascular endothelial growth factor (VEGF). With respect to the aforementioned molecules, this article reviews their pathophysiological importance, and puts the stress on whether they confer additional risk for invasive breast cancer or not. This knowledge has the potential to be of importance in the therapeutic decisions presenting in the common clinical practice.

## Background

Precursors and preinvasive lesions of the breast, which include atypical ductal hyperplasia (ADH), ductal carcinoma in situ (DCIS), and lobular neoplasia (LN), represent an heterogeneous entity with a lot of problems associated with the definition, classification, diagnosis and management of patients [1-5]. The diagnosis of these lesions represents a clinical dilemma for the patient and the physicians [2,3,5]. Following a diagnosis of atypical hyperplasia or DCIS, a patient is immediately considered at high risk for future development of invasive breast carcinoma, although this progression will only occur in a portion of patients [6,7]. Uncertainties in the prognosis have given rise to a debate surrounding the appropriate treatment, involving a wide range of treatment approaches from observation to mastectomy. This has resulted in diverse and confusing clinical recommendations, distressing to both patients and clinicians [3,8,9].

The use of molecular markers in the common clinical practice seems promising for the diagnosis and prognostication. Molecular markers nowadays seem to have the potential to improve our ability to care for patients with, or at risk for, breast cancer.

The aim of this review article is to describe the current theories regarding the pathogenesis and molecular evolution of preinvasive breast lesions. With respect to the molecules implicated, this article focuses especially on whether they confer additional risk for invasive breast cancer or not. The evaluation of molecular markers is performed under the light of their potential usefulness in treatment decisions presenting in the common clinical practice.

### Precursors and preinvasive lesions of the breast

ADH represents a proliferative lesion that fulfils some, but not all the criteria for a diagnosis of low-grade, non-comedo type DCIS [2,10]. In essence, ADH is usually small and focal, measuring less than 2 to 3 mm. ADH is a rare condition, being seen in 4% of symptomatic benign biopsies [1]. The significance of the diagnosis of ADH lies in the increased risk of subsequent invasive breast carcinoma (relative risk RR = 4.4) [10]. When ADH is combined with a positive family history, the relative risk of invasive cancer reaches 9.7 [11,12]. The major problem with regard to ADH is the difficulty in achieving acceptable levels of concordance or consistency in diagnosis. There is significant inter-observer [13] variability in the diagnosis of ADH. It should also always be born in mind that proliferation at the edge of a biopsy may represent the periphery of a more established lesion of DCIS [1].

DCIS is defined as a proliferation of malignant epithelial cells within the breast parenchymal structures with no evidence of invasion across the basement membrane [1]. Presently, DCIS constitutes 15–20% of screen-detected malignancies of the breast [14,15], and it is known that such a diagnosis confers an 8-10-fold elevated risk for the development of IBC [16]. Studies suggest that up to 50% of patients with microscopic foci of DCIS develop invasive carcinoma [1,6,7]. Additionally, it has been shown that progression to invasion is related to the subtype of DCIS; comedo disease progresses into invasive carcinoma both more often and more rapidly than low-grade DCIS [1,17].

LN is characterized by the proliferation of generally small and often loosely cohesive cells. The term LN refers to the entire spectrum of atypical epithelial proliferations originating in the terminal duct-lobular unit (TDLU), with or without pagetoid involvement of terminal ducts [2,4]. The designations ALH and LCIS, have been widely used for varying degrees of the lesions [2,18,19]. LN is a "marker of increased risk" rather than a true precursor of invasive carcinoma. ALH confers a 3-fold elevated risk for the development of IBC, while LCIS has a relative risk equal to 7 [2,20]. LN is multicentric in as many as 85% of patients and bilateral in 30% of women who had undergone bilateral mastectomy [2,4,19,20].

### Histological models of the progression to invasive carcinoma

Over the past twenty years, a histological model of human breast cancer evolution was predominant [21,22]. The multistep model of breast carcinogenesis supports a transition from normal epithelium to invasive carcinoma via non-atypical hyperplasia, atypical hyperplasia and in situ carcinoma [4,23,24]. Confirmatory evidence that precursors and preinvasive lesions are clonal processes arises from studies showing similar genetic changes in low-grade DCIS and ADH (Table 1), and identical genetic abnormalities with synchronous ipsilateral invasive breast cancer [4,25-34]. These studies were supported by mouse mammary tumor models [35] and by epidemiological studies [36] which showed that the risk for breast cancer increased with the rate of proliferation and atypia in breast biopsies. The fact that the invasive carcinoma occurs in the same area as the original indicates a precursor progress [1,39].

**Table 1:** Genetic predisposition in ADH, LN, DCIS.

**Genetic predisposition**		
	Losses	Gains

ADH:	1q, 2p, 6q, 9p, 11p, 11q, 13q, 14q, 16q, 17p, 17q, Xq	Unknown
ALH:	11q, 16p, 16q, 17p, 22q	6q
DCIS:	1p, 7q, 2p, 2q, 3p, 3q, 4p, 6p, 6q, 7p, 7q, 8p, 8q, 9p, 11p, 11q, 12p, 13q, 14q, 15q, 16p, 16q, 17p, 17q, 18q, 21q	1q, 3q, 6q, 6p, 8q, 17q, 20q, Xq
LCIS:	11q, 13q, 16p, 16q, 17p, 17q, 22q	6q

On the other hand, the lack of direct evidence for the linear progression model gave birth to new models. Farabegoli et al. [40] proved that DCIS is a possible, but not an obligate precursor of invasive breast cancer and suggest that pure DCIS and DCIS associated with IDC may be genetically distinct. The evolution from DCIS to IDC may thus follow multiple pathways rather than a linear model. Leong et al. suggested that in most cases, low-grade DCIS is associated with low-grade invasive carcinoma and high-grade DCIS with high-grade invasive carcinoma, i.e., horizontal progression. They postulated that intermediate-grade DCIS is heterogeneous and it is therefore possible that this group represents some cases of DCIS that have progressed from low-grade DCIS as well as cases that may progress to high-grade DCIS, suggesting that progression may not be entirely horizontal in intermediate-grade DCIS [39-43].

### Classification

All cases of ADH, LN, and DCIS do not have the same possibility to progress to invasive carcinoma [1,6,7]. It became thus obvious that a uniform approach is incorrect, and a subclassification arose. However, there is no specific classification for ADH and LN, while for DCIS the Van Nuys classification is the most widely accepted method for risk estimation and has replaced the Holland, the Bellamy, the Leal and the Lagios classifications [39]. The Van Nuys Prognostic Index (VNPI) combines three significant predictors of local recurrence: tumor size, margin width, and pathologic classification (nuclear grade, comedo-type necrosis). According to the VNPI, DCIS is classified into three groups with low, intermediate, or high risk of local recurrence after breast conserving therapy [44].

### Molecular markers

It has become apparent that the existing classification systems of precursors and preinvasive lesions are not adequate. The challenge lies in using available clinical and pathologic data to estimate the individual relative risk for invasive breast carcinoma rather than the relative risk of each lesion. This could be helpful for the selection of the most appropriate therapy for each individual patient. In the following pages, the available data with respect to important molecules are presented in detail. Table 2 summarizes the main features regarding the presented molecules.

**Table 2:** Molecular markers and their prognostic value in precursors and preinvasive breast lesions.

**Molecular markers**	**Prognostic importance**
Estrogen receptor-alpha	Aggravating, 3-fold increased risk for IDC (normal breast epithelium)
Estrogen receptor-beta	Protective
c-erb-B2	Aggravating -not unanimous results with respect to RR
p53 disruption	Aggravating – conflicting results regarding the RR of its expression in benign lesions
Ki-67	Aggravating – no specific estimation of RR
Bcl-2	Aggravating – no specific estimation of RR
VEGF	Aggravating
E-cadherin disruption	Aggravating
TGF- beta	Ambiguous – loss of TGF-beta-RII is aggravating
p16 disruption	Controversial findings
p21 (Waf1)	Controversial findings
p27 disruption	Aggravating/scarcity of data
14-3-3 sigma hypermethylation	Aggravating

#### Estrogen receptors (ER)

Estrogens play a central role in the growth and differentiation of normal breast epithelium, stimulating cell proliferation and regulating the expression of other genes, including the progesterone receptor (PgR) [45-47]. In the normal pre-menopausal breast, ER(+) cells comprise the 7% of the total epithelial cell population [48]. ER(+) cells are luminal epithelial cells, evenly distributed, and seem to secrete factors which paracrinely influence the proliferation of the adjacent ER(-) cells [48,49]. It should be noted that ER positivity and proliferation (as depicted by ki-67 expression, see below) are almost mutually exclusive in normal epithelial breast tissue [49]. ER+ cells increase with age, reaching a plateau after menopause [50].

The intensity of ER expression in the normal epithelium is a risk factor for breast cancer, conferring a 3-fold increase in risk [51]. More specifically, regarding non-atypical epithelial hyperplasia, ER positivity together with ki-67 expression, seem to make an important distinction between lesions: the existence of ki-67(+)/ER(+) cells seems to correlate with progression to more severe lesions [52]. Alternatively, it has been suggested that an increased percentage of ER(+) cells in the adjacent normal lobules seems to be associated with elevated risk for invasive breast cancer rather than ER-positivity within the lesion *per se *[53].

In other benign breast lesions, such as sclerosing adenosis, radial scars, papillomas, fibroadenomas, and phylloides tumours, the percentage of ER(+) cells is higher than normal breast tissue [54]. Similarly, ER-alpha expression has been proven significantly elevated in the hyperplastic enlarged lobular unit (HELU), which represents the earliest histologically identifiable lesion with premalignant potential. On the contrary, as far as enlarged lobular units with columnar alteration (ELUCA) are concerned, an intriguing result emerged, since more intense ER-alpha staining has been associated with a lower risk of subsequent invasive carcinoma in these lesions [55].

In the context of ADH, LN and DCIS, and contrary to the normal breast, ER(+) cells are surrounded by contiguous cells characterized also by ER-positivity [49]. Moreover, in DCIS, the above explained diptych ER(+)/Ki-67(+) is a hallmark [54]. In general, non-comedo carcinomas exhibit more frequently ER positivity [56,57].

In parallel, the less studied ER-beta, seems also to be of importance and to exhibit an inverse pattern to that of ER-alpha, declining during the progression from normal breast tissue to ADH, DCIS, and IDC [58,59]. Of notice, according to a recent article, a high ratio ER-alpha/ER-beta in non-atypical epithelial hyperplasia seems to predict progression to carcinoma [60]. Finally, for the optimal envisagement of the ER network, it should be kept in mind that important regulators exist, such as hsp-27 [61] or AIB1 [62], exhibiting more intense expression in breast cancer.

#### Progesterone receptors (PR)

Similarly to estrogen receptors, progesterone receptors (PR) have been found elevated very early in premalignant breast lesions, at the hyperplastic enlarged lobular unit (HELU) [63]. On the other hand, a decreasing trend for PR expression has been documented along with progression to malignancy [64]. In DCIS, PR positivity is associated with ER positivity and lack of comedo necrosis [65-67]. Concerning PR positivity, studies are contradictory with respect to tumor grade [65,66,68,69] and recurrence rate [[70-72], reviewed in ([72])]. In IDC, progesterone receptors have been associated with histological grade, but not with lymph node involvement, tumor size, or prognosis of the patients [64]. With respect to lobular neoplasia, there is scarcity of data; PR seem however to be expressed in the majority of cases [74,75].

More interestingly however, it is the ratio progesterone receptor A (PRA)/progesterone receptor B (PRB) which seems to play a central role. In normal breast tissue and non-atypical hyperplasia, receptors are homogenously coexpressed, but early during progression, one receptor (especially PRA in advanced lesions) predominates at a heterogeneous manner [76]. Indeed, the physiological role of PRA predominance has been supported by in vitro studies, demonstrating its modulating effects on cell morphology and adhesion [77,78]. Of notice, in the normal tissue of BRCA mutation carriers, PRB isoform is strikingly absent [79].

#### C-erbB-2 (Her-2/neu)

HER-2/neu, a gene located on 17q, encodes for c-erbB-2 oncoprotein, a tyrosine kinase receptor. Alterations of c-erbB2 (HER-2/neu) are suggested to be an important event in malignant transformation [80-82]. According to a variety of studies, c-erbB-2 has not been found overexpressed at the protein level in benign proliferative breast disease or ADH [83-86]. However, amplification of Her-2/Neu has been documented with the use of FISH in ADH, supporting the notion that the degree of HER-2/neu amplification increases with progression to carcinoma [87]. Accordingly, patients with benign breast lesions showing low levels of amplification of the HER-2 gene have a two-fold increased risk of breast cancer [88]; however, according to another study, c-erbB-2 overexpression in benign lesions was not a significant risk factor [89].

With respect to lobular neoplasia, one fourth of LCIS cases have been found positive for c-erbB-2, irrespectively of the coexistence of an invasive component [90]. Occasional positivity has been found also in pleomorphic lobular (ductal-lobular carcinomas in situ [91].

As far as the role of c-erbB-2 in DCIS is concerned, C-erbB-2 immunoreactivity has been primarily associated with DCIS of higher grade, in the absence [63] or presence [92] of IDC, and with comedo type [93]. Interestingly, given the association of higher grade with c-erb-B2 amplification, the latter has been regarded as an independent prognostic factor [94]. Allred et al [95] documented that the percentage of c-erb-B2 immunoreactivity is significantly higher in DCIS than IDC: one of the possible explanations the authors gave was that c-erb-B2 may be more important for the initiation than the progression of breast cancer, or that c-erb-B2 may be downregulated during the progression of breast cancer.

#### p53

P53 is a tumour suppressor gene located on 17p. p53 protein mediates its tumor suppressor functions via the transcriptional regulation or repression of a variety of genes [96-98] and is an important component of breast cancer pathophysiology [99]. Regarding the role of p53 as a risk factor in benign breast lesions, there is controversy of data: the immunohistochemical detection of p53 in benign breast lesions has been associated with elevated cancer risk [89], although there are studies with conflicting results [100].

Considering the various types of lesions in the continuum between benign lesions and breast cancer, various studies have assessed the role of p53. In epithelial hyperplasia without atypia, p53 mutations have not been detected [101]. In ADH, the presence and role of p53 mutations is still an open field: p53 mutations were initially not documented [102]; then, studies pointing to p53 mutations appeared [103], and, more recently, the presence of mutated p53 in ADH has been demonstrated with the use of laser capture microdissection microscope, single-stranded conformational polymorphism (SSCP) and sequencing [104]. Regarding LN, there is scarcity of data: in two studies, no p53 immunoreactivity was demonstrated in LN lesions [105,106], whereas a more recent one on LCIS reported p53 immunoreactivity in one fifth of cases [90].

p53 mutations/accumulation are present in a significant percentage of DCIS [107-111], especially in the comedo type [112]. However, the clinical significance of p53 accumulation remains still elusive; although it has been found to influence the proliferation rate [113], a recent study showed that it does not affect the proliferation rate of the DCIS lesion *per se *[107]. Of notice, the coexistence of DCIS with IDC is not associated with a different degree of p53 immunostaining [114].

#### Ki-67

Ki-67 is a cell cycle-associated nuclear protein, which is expressed in all cycle phases, with the exception of G0 and early G1, and reacts with MIB-1 antibody [115]. Protein Ki-67 is extensively used as a proliferative index and is linked with malignancy, even in FNA specimens [116]. Moreover, its intrinsic association with apoptosis (bcl-2 status, see below) and p53 expression (see above) seems to be of importance in the diagnosis and prognosis of precursors and preinvasive breast lesions: low Ki-67 expression/bcl-2 positivity and p53 negativity are a trait of ADH and, subsequently, well-differentiated carcinomas. On the contrary, high Ki-67 expression/bcl-2 negativity within the lobules implicate lesions with a potential of poorly differentiated carcinoma [117]. As mentioned above, also in the context of non-atypical hyperplasia, high Ki-67 and ER-alpha expression seem to predict progression to cancer [51,118].

Interestingly enough, a clinical application of Ki-67 expression intensity seems to emerge. In non-atypical ductal hyperplasia, lesions with high Ki-67 expression can be clinically detected scintimammographically, since high (99m)Tc-(V)DMSA uptake seems to be their feature. According to the authors, this could prove useful in identifying women with benign but high-risk breast pathologies [119].

#### Bcl-2

The bcl-2 gene is located on 18q. Bcl-2 protein, belongs to a family of proteins playing a central role in the regulation of apoptosis [reviewed in ([120]), [121,122]] and other pathways [reviewed in ([123])]. With respect to the overall role of apoptosis in breast cancer pathogenesis, there seems to be an intriguing pattern incorporating the proliferation of the lesion. Growth imbalance in favour of proliferation seems crucial in the transition from normal epithelium to hyperplasia and later, from preinvasive lesions to IDC. On the contrary, apoptosis becomes more important at an intermediate stage: in the transition from hyperplasia to preinvasive lesions, the imbalance is in favour of apoptosis [124]. Bcl-2 is present in the whole spectrum of breast lesions: predominantly in benign lesions, ADH, LN, and well-differentiated DCIS [105,125-127]. More specifically, there is a gradual increase in the extent of apoptosis [124,128] and a parallel decrease in bcl-2 expression in benign/precursors/preinvasive/invasive lesions as they become histologically more aggressive [128]. Bcl-2 positivity tends to coincide with p53 negativity in normal breast tissue, non-atypical ductal hyperplasia, ADH, LN and in the majority of the DCIS [105]. The role of Bcl-2 expression as a risk factor for breast cancer is described above, together with Ki-67 (see above).

#### Vascular endothelial growth factor (VEGF) and angiogenesis

VEGF is a potent angiogenic growth factor, commonly involved in tumor-induced angiogenesis, with a putative therapeutical significance in the context of breast cancer [129]. Of notice, VEGF gene polymorphisms have been associated with modified breast cancer risk in various populations [130,131].

Viacava et al have thoroughly examined the angiogenesis in precursor and preinvasive lesions. Increased vascularization is present in all preinvasive lesions and increases with lesion severity. In ductal lesions, angiogenesis is more intense in poorly/intermediately differentiated intraductal carcinomas than in non-atypical ductal hyperplasia and ADH. Similarly, LCIS, showing microvascular density similar to that of poorly/intermediately differentiated intraductal carcinoma, is more vascularized than ALH. In the same study, VEGF expression in normal glandular structures was lower than in lesions, with the highest levels found in ductal lesions. Interestingly, no correlation was found between VEGF expression and the degree of vascularization in that study [132]. On the other hand, Hieken TJ et al. suggested that VEGF expression may help predict the biologic aggressiveness of DCIS [133]. Additionally, in the context of DCIS, Vogl et al support VEGF expression is not regulated by the HER2 pathway [134].

#### E-cadherin

E-cadherin, a tumor suppressor gene located on 17q, has been implicated especially in lobular breast cancer molecular pathogenesis [135]. In clinical practice, immunohistochemistry for E-cadherin is a helpful marker for differential diagnosis, since most cases of low-grade DCIS exhibit E-cadherin positivity, whereas LN is almost always E-cadherin negative [[136], reviewed in ([137]), ([138])]. This implies that E-cadherin disruption is an early event, prior to progression, in lobular carcinogenesis [139,140]; more specifically, DNA alterations accompanying the loss of protein expression pertain to LCIS but not to ALH [140]. As expected according to the above, only few studies have focused on E-cadherin in ductal lesions. In the context of DCIS, hypermethylation of E-cadherin 5' CpG islands has been demonstrated [141], and, at the protein level, E-cadherin has been linked to better differentiation [142]. Moreover, mutational analysis of E-cadherin provided evidence to support that DCIS is the precursor of invasive ductal carcinoma in cases where LCIS coexists [143].

#### TGF-beta

The transforming growth factor-beta (TGF-β) pathway has ambivalent importance in the pathogenesis of breast cancer [reviewed in ([144])]. Serum TGF-beta levels do not differ between patients with breast cancer, DCIS and benign lesions [145]; however, TGF-beta expression becomes more accentuated in IDC, compared with DCIS [146]. Surprisingly enough, an interesting study recently showed that loss of TGF-beta-RII expression in epithelial cells of hyperplasia without atypia is associated with increased risk of IDC [147]. No reports exist on ADH and LN, to our knowledge.

#### P16 (INK4a)

p16 is an inhibitor of cyclin-dependent kinases 4 and 6 [reviewed in ([148])]. With respect to the role of p16, controversial results exist. According to some authors, aberrant methylation of p16 is not demonstrated in benign conditions, epithelial hyperplasia and intraductal papillomas, but is restricted in cancerous epithelium [149]. On the contrary, another study showed that IDC demonstrate hypomethylation of p16 and hyperactivity of the p16 gene (enhanced expression of p16 mRNA), contrary to the hypermethylated, inactive state in the normal epithelium. [150]. Independently, Di Vinci et al. distinguish between p16 hypermethylation and p16 protein overexpression; the former seems not to be specifically associated with malignancy and to occur both in benign and malignant lesions, whereas the latter, together with cytoplasmic sequestration, is a feature of breast carcinoma. [151]. In the context of such controversy, no studies exist with respect to p16 as a risk factor, with the exception of a study in Poland envisaging p16 as a low penetrance breast cancer susceptibility gene [152].

#### p27 (Kip1)

The p27 gene encodes for an inhibitor of the cyclin – CDK (cyclin-dependent kinase) active complex, Although numerous studies exist with respect to the role of p27 in breast cancer [reviewed in ([153]), ([154]), ([155])], there is lack of data regarding precursors, preinvasive lesions and other predisposing conditions. p27 expression has been documented in DCIS, but its clinicopathological significance is still uncertain [156].

#### P21 (Waf1)

p21 is a cell cycle regulator, implicated in a variety of pathways [157]. p21 immunoreactivity has been detected both in benign and malignant epithelium, and thus its role is hard to interpret. [126]. Studies focusing especially on ADH, or LN do not exist. As far as DCIS is concerned, p21 positivity has been independently associated with clinical recurrence [158]. On the other hand, Oh YL et al. found a significant correlation between positive p21 immunoreactivity (67.3% of the cases) and well-differentiated histologic grade, non-comedo type, ER-positive and p53-negativity. According to them, DCIS with p21+/p53- is likely to be the non-comedo type [156].

#### 14-3-3 sigma

Umbricht et al identified 14-3-3 sigma as a gene whose expression is lost in breast carcinomas, primarily by methylation-mediated silencing. Importantly, the hypermethylation of the locus was absent in hyperplasia without atypia, but was detectable with increasing frequency as the breast lesions progressed from atypical hyperplasia to DCIS, and finally to invasive carcinoma [159]; of notice, methylated alleles existed in the periductal stromal breast tissue. Afterwards, a parallel, stepwise reduction at the 14-3-3 sigma protein level has been documented [160].

Despite the emerging role of 14-3-3 sigma in breast carcinogenesis, to date no studies exist assessing its role as a risk factor for breast cancer development.

### Therapeutic decision – perspectives

The evaluation of the molecules whose importance has been to date elucidated could be adjunctively useful in the therapeutic decision. A simultaneous presentation of more than one aggravating factors might detect preinvasive lesions at risk of progressing to malignancies and influence the clinical decisions, such as prophylactic surgery (lumpectomy versus mastectomy for excision of the diseased ducts before the development of invasive carcinoma), and chemo-prophylaxis.

For the future of the preinvasive breast lesions' management, it is tempting to anticipate the gradual integration of their molecular profiling in the clinical practice. The simultaneous evaluation of multiple genes has recently appeared for the detection of healthy individuals at risk for breast cancer [161] and will be of special interest also in the context of ADH or LN. An appropriate combination of techniques (immunohistochemistry, fluorescent in situ hybridisation, analysis of LOH, CGH, DNA microarrays proteomics analysis, etc) might be helpful in this direction.

## Conclusion

The clinician should be aware of recent progresses in molecular biology, individualising his approach to every patient based on her own risk factors, tumour markers, histological profile, psychological and social status, etc. With respect to the molecular markers presented above, this article reviews the progress made, but also the existing controversies which should be further studied. Indeed, the existing controversies are particularly significant, and point to the need for further research.

## Abbreviations

ADH: atypical ductal hyperplasia,

DCIS: ductal carcinoma in situ,

LN: lobular neoplasia,

R.R: relative risk,

IBC: invasive breast carcinoma,

TDLU: terminal duct-lobular unit,

VNPI: Van Nuys Prognostic Index,

ER: Estrogen receptors

HELU: hyperplastic enlarged lobular unit,

ELUCA: enlarged lobular units with columnar alteration,

PR: Progesterone receptors,

VEGF: Vascular endothelial growth factor

## Competing interests

The author(s) declare that they have no competing interests.

## Authors' contributions

Flora Zagouri: writing of the manuscript, conception of the idea, review of the literature

Theodoros N. Sergentanis: revised the manuscript, writing of the manuscript

George C. Zografos: revised the manuscript critically for important intellectual content, final approval of the version to be published.
